# Supplementary material for: Dynamics of self-hybridized exciton–polaritons in 2D halide perovskites
Source: Light Sci Appl. 2024 Jan 1;13:1. doi: 10.1038/s41377-023-01334-9 (PMC10757995; doi:10.1038/s41377-023-01334-9)
Supplement: Supplementary file 1 — Supplementary Information: Charge and Energy Transfer Dynamics in 2D Perovskite Polaritons [file 41377_2023_1334_MOESM1_ESM.pdf]

Supplementary Information

Dynamics of Self-Hybridized Exciton-Polaritons in 2D Halide Perovskites

Surendra B. Anantharaman[1,], Jason Lynch[1], Christopher E. Stevens[2, 3], Christopher Munley[4], Chentao Li[5], Jin Hou[6, 7], Hao Zhang[6, 7], Andrew Torma[6, 7], Thomas Darlington[8], Frank Coen[1], Kevin Li[1], Arka Majumdar[4,9], P. James Schuck[8], Aditya Mohite[5,6], Hayk Harutyunyan[5], Joshua R. Hendrickson[3], Deep Jariwala[1,*]*

[1] Department of Electrical and Systems Engineering, University of Pennsylvania, Philadelphia, Pennsylvania 19104, United States

[2]KBR Inc., Beavercreek, Ohio, 45431, United States

[3] Air Force Research Laboratory, Sensors Directorate, Wright-Patterson Air Force Base, Ohio 45433, United States

[4] Department of Physics, University of Washington, Seattle, Washington 98195, United States

[5] Department of Physics, Emory University, Atlanta, Georgia 30322, United States

[6] Department of Chemical and Biomolecular Engineering, Rice University, Houston, Texas 77005, United States

[7] Department of Materials Science and Nanoengineering, Rice University, Houston, Texas 77005, United States

[8] Department of Mechanical Engineering, Columbia University, New York, New York 10027, United States

[9] Department of Electrical and Computer Engineering, University of Washington, Seattle, Washington 98195, United States

Correspondence

Deep Jariwala (dmj@seas.upenn.edu)

[1] Department of Electrical and Systems Engineering, University of Pennsylvania, Philadelphia, Pennsylvania 19104, United States. Phone number: +1-215-746-4380

(or)

Surendra B. Anantharaman (sba@iitm.ac.in)

[1] Department of Electrical and Systems Engineering, University of Pennsylvania, Philadelphia, Pennsylvania 19104, United States. Phone number: +91 44 - 2257 4794

Enhancing environmental and excitation stability of 2D HOIP crystals supporting self-hybridized exciton-polaritons

The higher-order (HO) modes emerging from the strong coupling of exciton-polaritons can also serve as an optical sensor owing to the high Q and narrow linewidths that they possess which are extremely sensitive to the surrounding dielectric medium (See figure S6). Monitoring perovskite quality in-situ during optical measurements under high photon flux densities by shifting the excitonic peak is less sensitive due to broad linewidth (15 nm). Here we used the HO mode with ultranarrow linewidth (<3 nm) to monitor the perovskite degradation in ambient atmosphere by comparing two samples - pristine Au/RP2 flakes and polystyrene encapsulated Au/RP2 flakes. As seen in Figure S1, the pristine samples showed a drastic degradation in HO mode by 67% within 24 h. However, the encapsulated sample showed no change after 150 h exposure to ambient atmosphere.

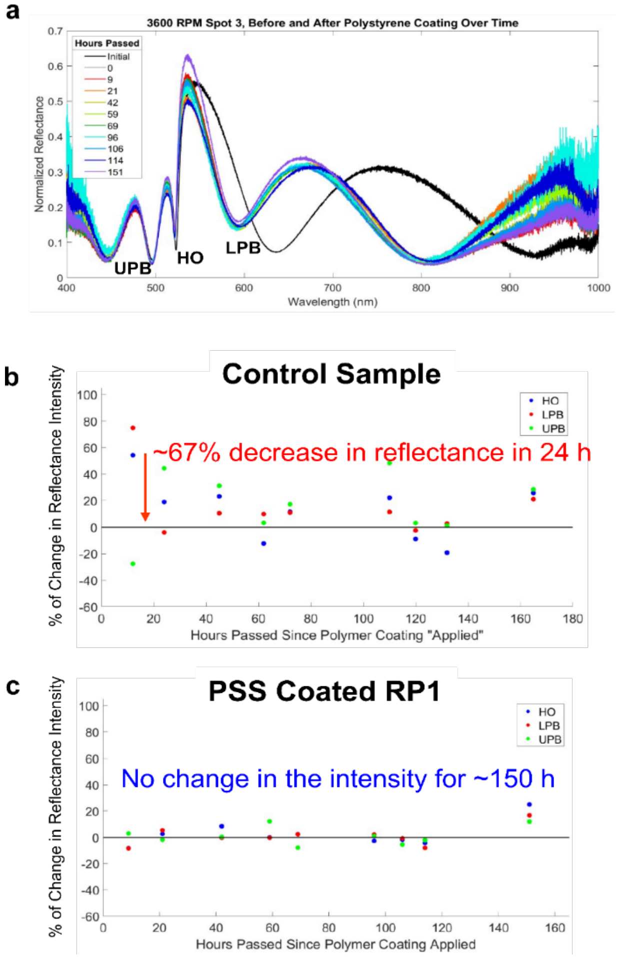

Figure S1. RP1 encapsulated in Polystyrene and HO mode sensor. (a) RP1 exfoliated on Au substrate forming HO mode was used as an optical sensor to monitor perovskite degradation. (b) Unencapsulated RP1 shows a 67% drop in the perovskite absorption for the HO mode. (c) polystyrene encapsulated RP1 shows no change in the intensity for 150 h in ambient atmosphere, thereby confirming the enhanced stability at room temperature and validating the HO mode as an optical sensor.

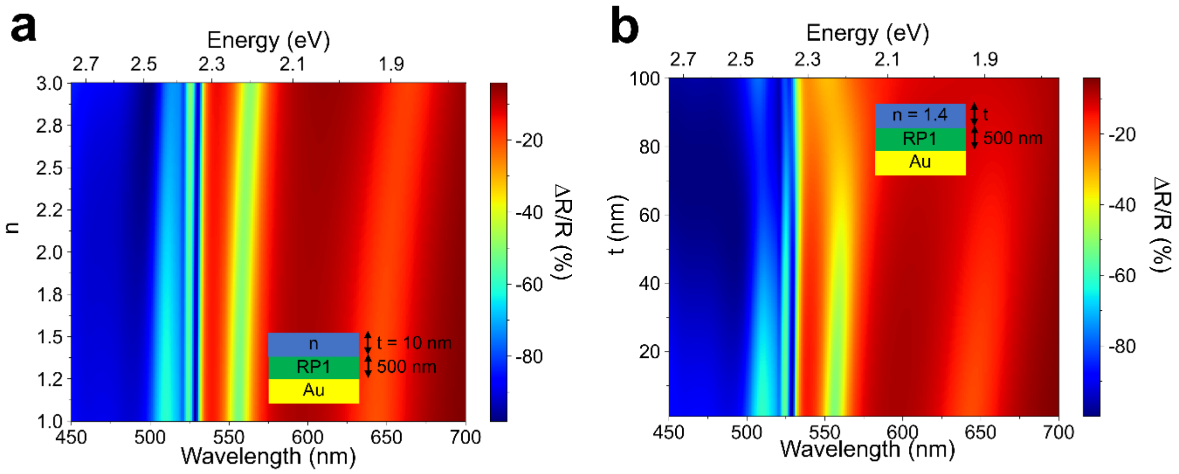

Figure S2. Sensing using RP1 The normalized change in reflectance for (a) a film of varying refractive index, n, that is 10 nm thick on top of 500 nm of RP1 and an Au substrate, and (b) a film of with a refractive index of 1.4 and varying thickness, t, on top of 500 nm of RP1 and an Au substrate. A refractive index of 1.4 was chosen for (b) since it is comparable to most organic molecules.

Table S1. Q-factor of the experimental UPB, HO, and LPB in the thick film (>500 nm) as well as uncoupled excitons in thin films perovskites. The UPB and LPB are the two modes closest to the HO. The Q-factor for the UPB, HO, and LPB was calculated using the reflectance spectra. The Q-factor for the uncoupled exciton is from the fit parameters of our previous work[1].

	UPB	HO	LPB	Exciton
RP1	12.0	131.6	78.6	54.0
RP2	8.6	101.4	52.7	39.4
RP3	7.3	82.3	38.0	33.4

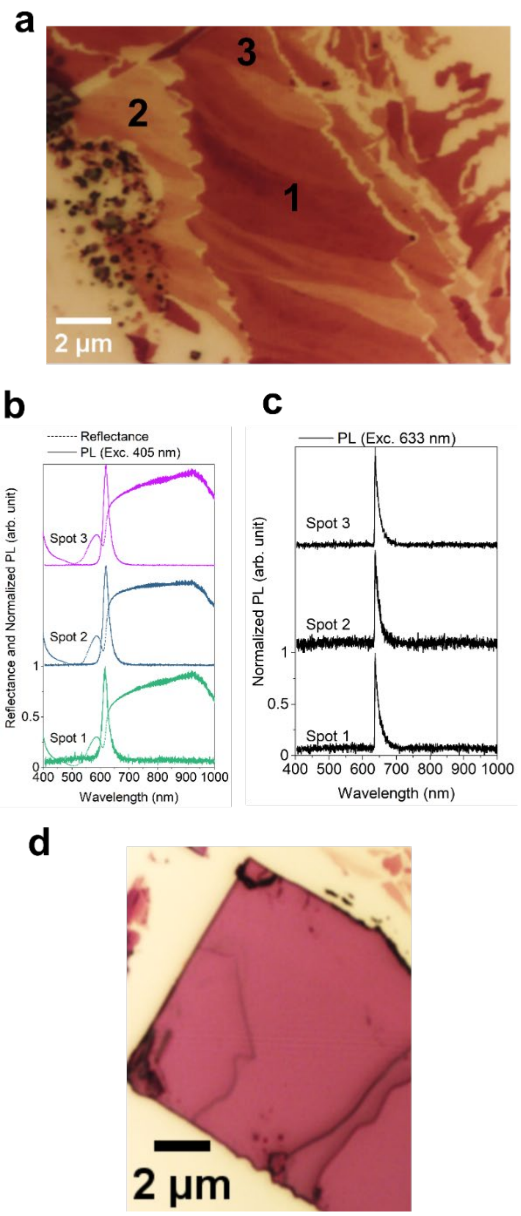

Figure S3. Exciton emission in RP3 without layer-edge state (LES) emission. a) Optical image of an exfoliated thin RP3 flakes on the Au substrate with three different thicknesses marked as 1-3. Photoluminescence spectra were recorded at room temperature from spots 1-3 with 405 nm (b), and 633 nm (c) pump laser. No LES emission was observed from thin RP3 flakes. In panel c, the broad signal up to 660 nm is due to the tail emission emerging from the weak excitonic absorption at 633 nm (excitation wavelength). d) Optical image of the thick RP3 flake exfoliated on the Au substrate.

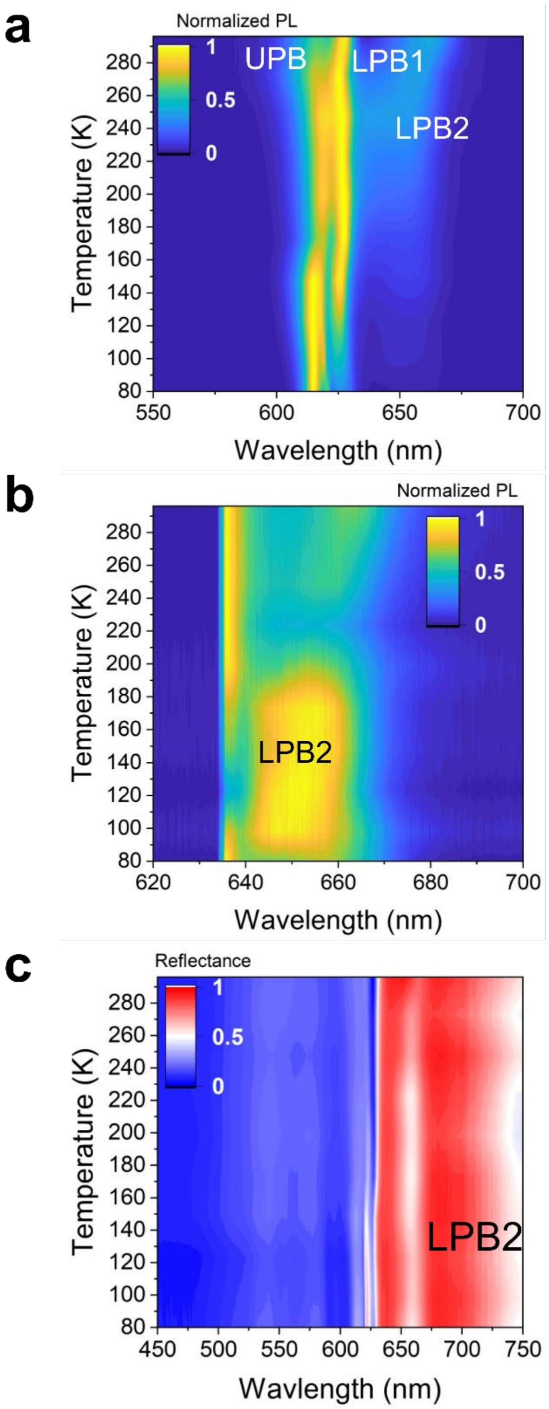

Figure S4. Temperature-dependent exciton-polariton dynamics in RP3 on Au. The normalized PL for excitation at 405 nm (a) and 633 nm (b), shows the presence of LPB2 emission at low temperature. Increase in LPB2 absorption at low temperature is clear from the reflectance studies (c).

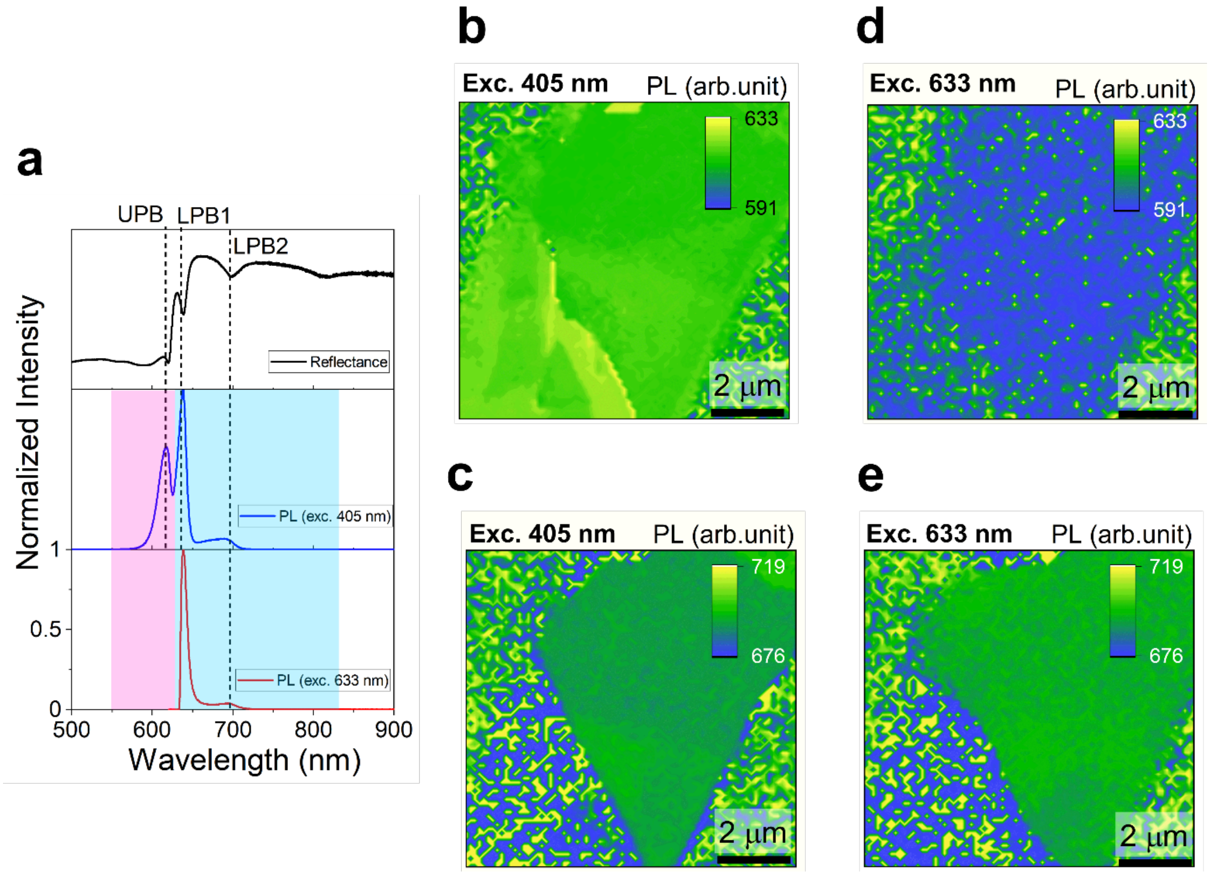

Figure S5. Room-temperature PL mapping from exciton-polaritons in RP3 exfoliated on the Au substrate. (a) Reflectance, PL from 405 nm and 633 nm excitation wavelength from RP3 exfoliated on Au. PL mapping for 405 nm excitation showing the presence of UPB and HO (b) and multiple LPB modes (LPB1 and LPB2) (c). PL mapping for 633 nm excitation showing the absence of UPB and HO (d) and presence of multiple LPB modes (LPB1 and LPB2) (e).

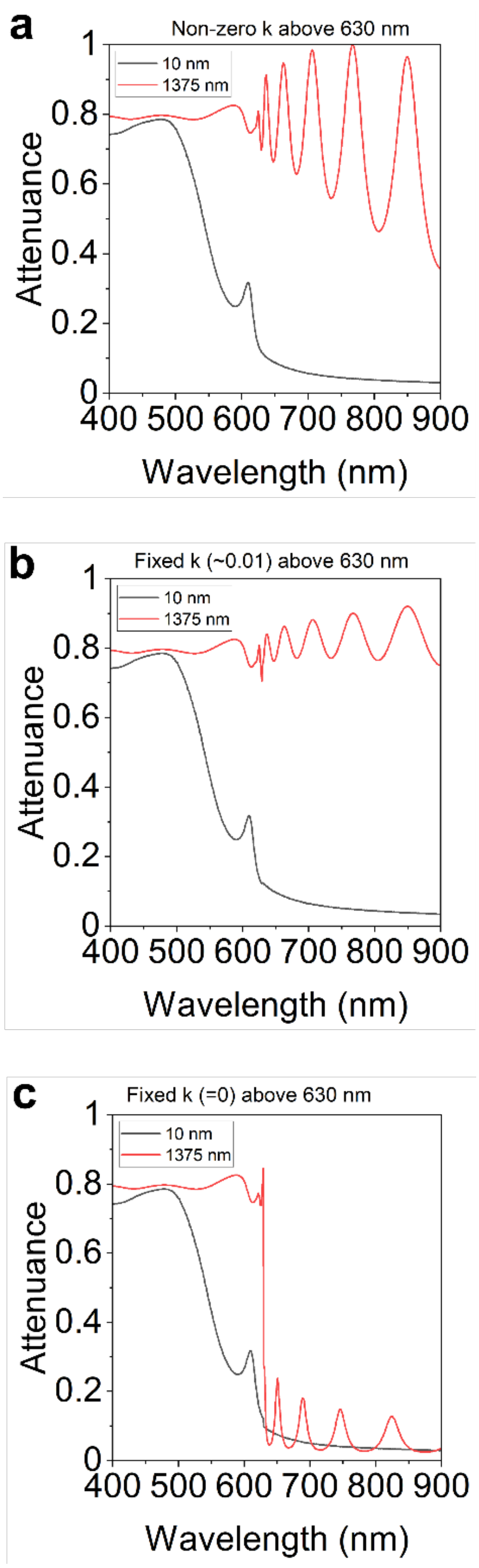

Figure S6. Attenuance spectra with varying k above bandgap for RP3. The attenuance spectra calculated for thin flakes (10 nm) and thick flakes (1375 nm) for a non-zero k (a), k = 0.01 (b) and k = 0 (c).

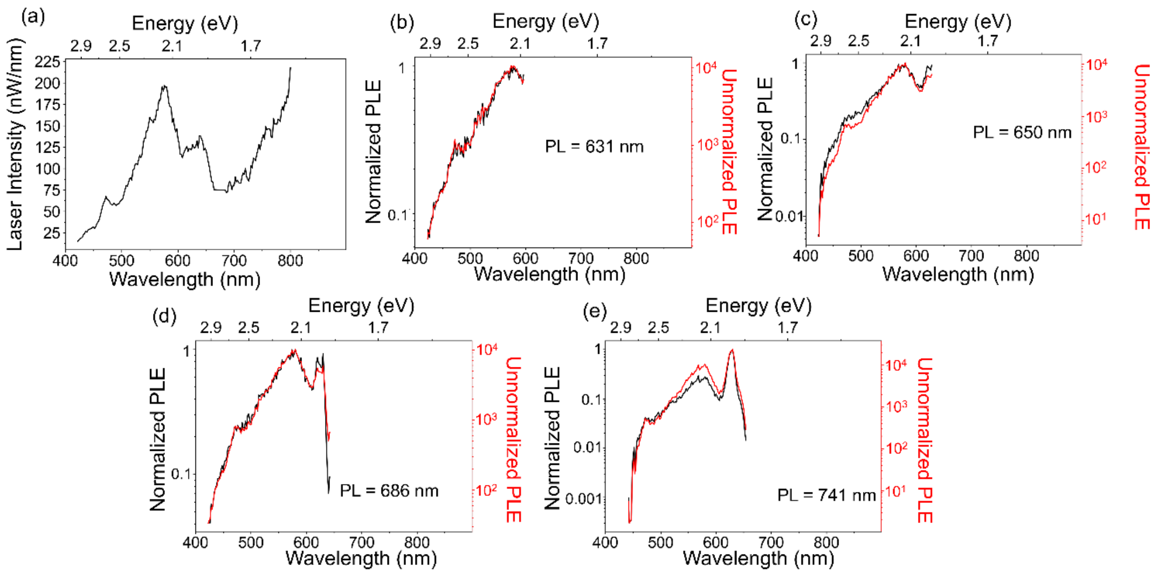

Figure S7. PLE data. (a) The wavelength dependent intensity of the laser used to perform PLE measurements. The normalized and unnormalized PLE spectra for emission at (b) 631 nm, (c) 650 nm, (d) 686 nm, and (e) 741 nm.

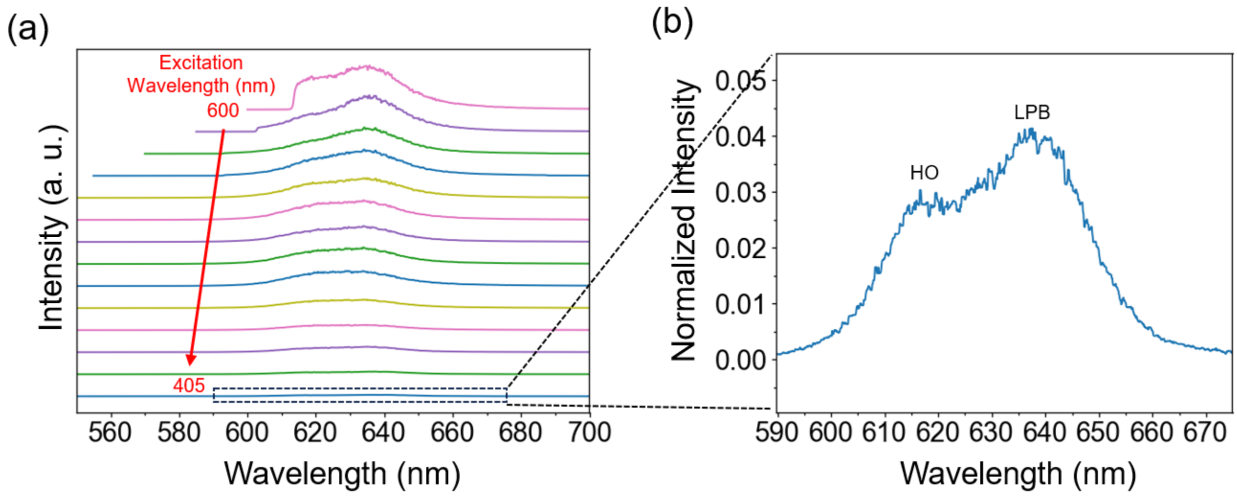

Figure S8. PLE of RP3 (104 nm) on Au at 300 K. (a) Photoluminescence (PL) spectra of RP3 (104 nm) on Au with excitation wavelengths varying from 405 nm to 600 nm with a step size of 15 nm. (b) The PL spectrum with an excitation wavelength of 405 nm normalized to the maximum intensity of the 600 nm excitation PL spectrum. The LPB was found to be 4% as emissive when excited with 405 nm light as

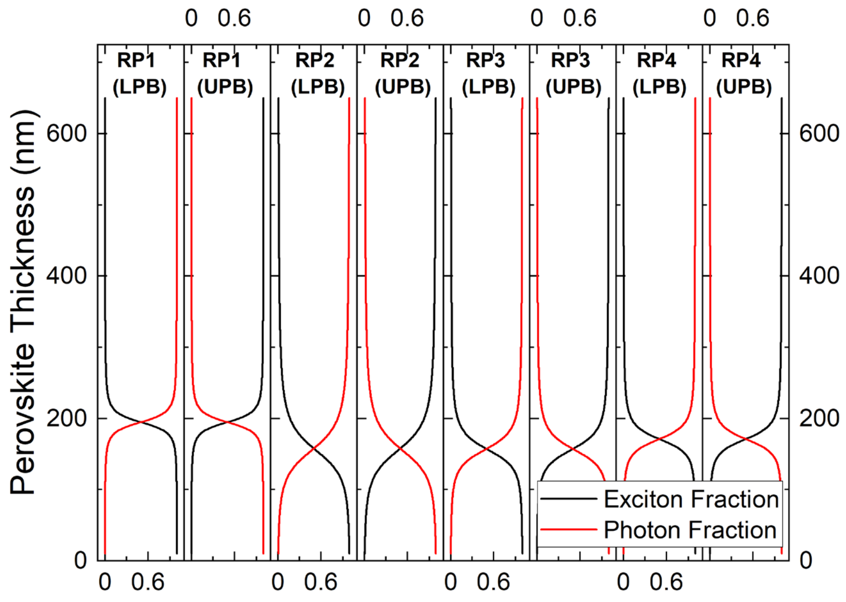

Figure S9. Hopfield coefficients. With increasing perovskite thickness, the Hopfield coefficient which provides the fraction of exciton and photon content in the UPB and LPB are shown here.

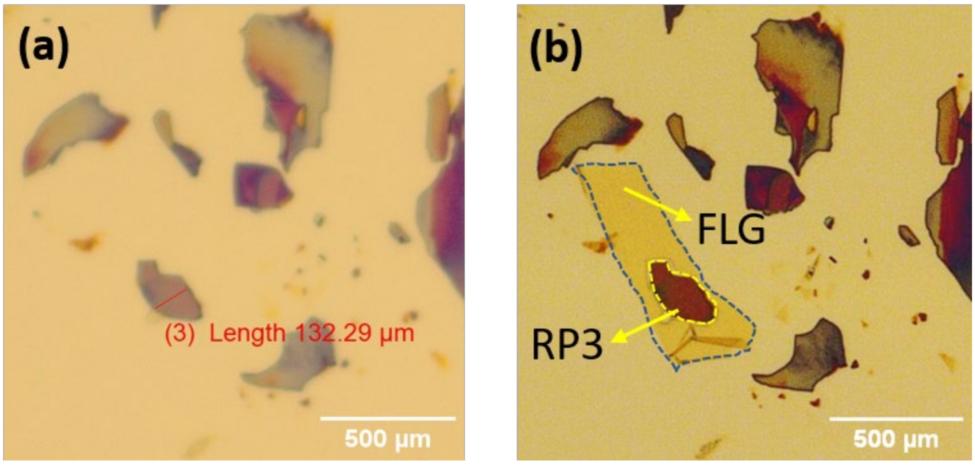

Figure S10. Optical micrograph of Au/RP3. (a) Exfoliated RP3 flakes on the Au substrate (b) Au/RP3/FLG heterostructure used for characterizing the reflectance and PL data shown in Figure 5e and 5f (main text).

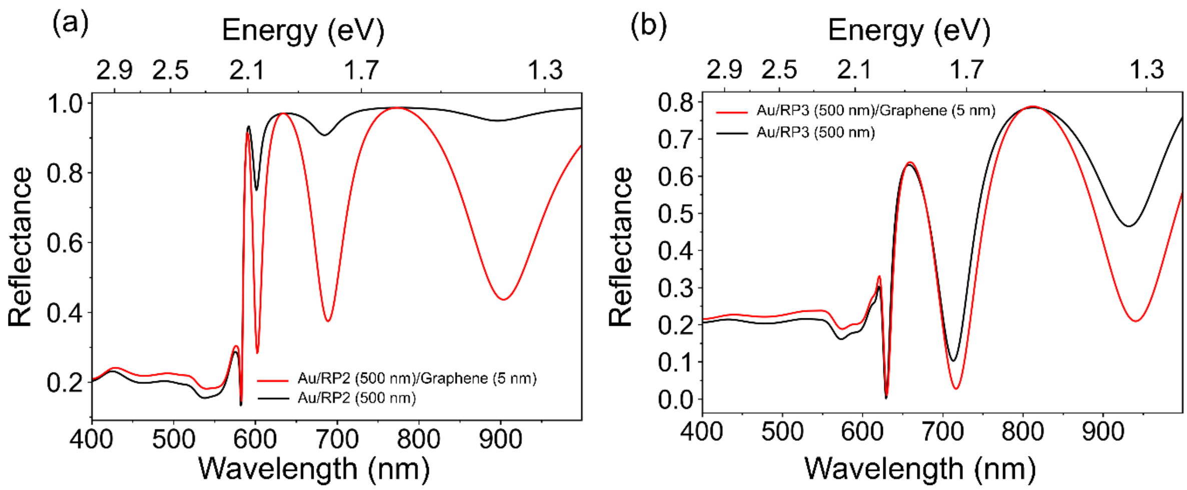

Figure S11. Simulated Reflectance Spectra of Heterostructures. Transfer matrix calculated reflectance spectra for (a) RP2 and (b) RP3 with and without few-layer graphene. The perovskite thicknesses were chosen to be 500 nm to show the presence of multiple LPBs.

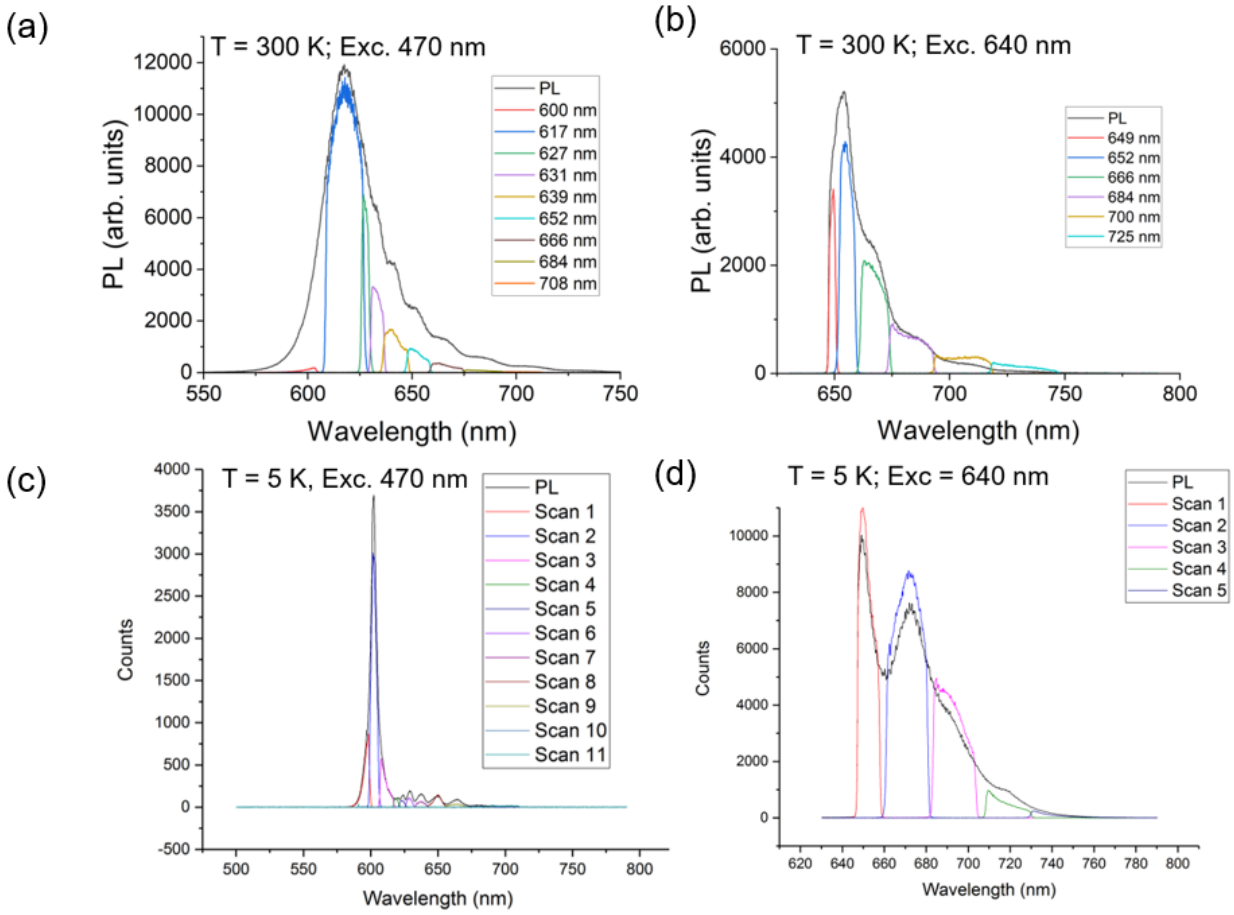

Figure S12. Filtered Photoluminescence (PL) Spectra of RP3 (610 nm) on Au at room and cryogenic temperatures. PL spectra of RP3 (610 nm) on Au at 300 K with excitation wavelengths of (a) 470 nm and (b) 640 nm along with PL spectra measured with filters whose central wavelengths are given in the legends. Upon cooling to 5 K, the measurement are repeated with excitation wavelengths of (c) 470 nm and (d) 640 nm.

References

1. Song, B. *et al.* Determination of Dielectric Functions and Exciton Oscillator Strength of Two-Dimensional Hybrid Perovskites. *ACS Mater Lett* **3**, 148–159 (2021).
2. Blancon, JC. *et al.* Scaling law for excitons in 2D perovskite quantum wells. *Nat Commun* **9**, 2254 (2018).
